# Spatially similar surface energy flux perturbations due to greenhouse gases and aerosols

**DOI:** 10.1038/s41467-018-05735-y

**Published:** 2018-08-14

**Authors:** Geeta G. Persad, Yi Ming, Zhaoyi Shen, V. Ramaswamy

**Affiliations:** 1 Department of Global Ecology, Carnegie Institution for Science, Stanford, 94305 CA USA; 2 Program in Atmospheric and Oceanic Sciences, Princeton University, Princeton, 08540 NJ USA; 3 NOAA Geophysical Fluid Dynamics Laboratory, Princeton, 08540 NJ USA

## Abstract

Despite distinct geographic distributions of top-of-the-atmosphere radiative forcing, anthropogenic greenhouse gases and aerosols have been found to produce similar patterns of climate response in atmosphere-and-ocean coupled climate model simulations. Understanding surface energy flux changes, a crucial pathway by which atmospheric forcing is communicated to the ocean, is a vital bridge to explaining the similar full atmosphere-and-ocean responses to these disparate forcings. Here we analyze the fast, atmosphere-driven change in surface energy flux caused by present-day greenhouse gases vs aerosols to elucidate its role in shaping the subsequent slow, coupled response. We find that the surface energy flux response patterns achieve roughly two-thirds of the anti-correlation seen in the fully coupled response, driven by Rossby waves excited by symmetric changes to the land–sea contrast. Our results suggest that atmosphere and land surface processes are capable of achieving substantial within-hemisphere homogenization in the climate response to disparate forcers on fast, societally-relevant timescales.

## Abstract (web summary)

Anthropogenic aerosol and greenhouse gas concentrations are spatially uncorrelated, but the climate response to each shows a similar spatial pattern. Here the authors show that two-thirds of the spatial similarity in the full response manifests through fast-acting atmosphere and land surface processes alone.

## Introduction

Although it has been demonstrated that the fully coupled response to greenhouse gases (GHGs) and aerosols have significant, though not complete, spatial pattern similarity, the mechanisms for pattern formation remain poorly characterized^1–3^ suggest that the spatial similarities in the fully coupled response to GHGs and aerosols, analyzed in a subset of the Coupled Model Intercomparison Project Phase 5 (CMIP5) models, are strongly mediated by common patterns of ocean–atmosphere feedbacks that can be separated conceptually into a fast, atmosphere-only component and a slower, ocean–atmosphere coupled component. The degree of spatial similarity in the climate response to GHGs vs aerosols has significant implications for questions in the detection and attribution of anthropogenic climate change^4^ and for our understanding of the transient climate response to heterogeneous vs homogeneous forcers^5^. It is thus vital to characterize the fast and slow mechanisms via which the spatial patterns of response are produced.

We probe the fast, atmosphere-and-land-only component of the formation of the spatial patterns of response to present-day GHGs and aerosols in an atmospheric general circulation model—a crucial intermediate step toward understanding the fully coupled response. We find that, although the atmospheric distributions of the two forcers are largely uncorrelated, their surface energy flux perturbation patterns achieve roughly two-thirds of the anti-correlation seen in the fully coupled response. Rapid land temperature and precipitation responses also show strong pattern similarity. Our analysis highlights the common modes of atmospheric circulation and surface energy adjustment that are triggered by both GHG and aerosol forcings. These produce antisymmetric (i.e., symmetric, but of opposite sign) spatial patterns of surface sensible and latent heat flux variations in response to the two forcers, particularly over the summertime Northern Hemisphere oceans. Our results suggest that atmosphere and land surface processes are capable of achieving substantial within-hemisphere homogenization in the climate response to disparate forcers on fast timescales, with implications for detection and attribution of near-term climate change and for understanding and predicting the regional climate impacts of anthropogenic GHGs and aerosols on societally-relevant timescales.

## Results

### Aerosol and greenhouse gas radiative forcing patterns

**Fig. 1:**
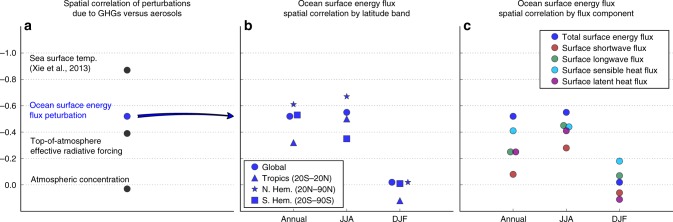
Spatial correlations of perturbations due to greenhouse gases vs aerosols. **a** The spatial correlation of the ocean-masked surface energy flux perturbation due to greenhouse gases vs aerosols (blue dot) is substantially greater than that of the atmospheric concentrations of the two forcers or the top-of-atmosphere effective radiative forcing, and achieves approximately two-thirds of the spatial correlation in the eventual sea surface temperature response^3^. Decomposition of the ocean surface energy flux pattern correlation seasonally by latitude band (**b**) and by surface energy flux component (**c**) shows that the spatial correlation manifests most strongly in the surface heat fluxes over the summertime Northern Hemisphere oceans

**Fig. 2:**
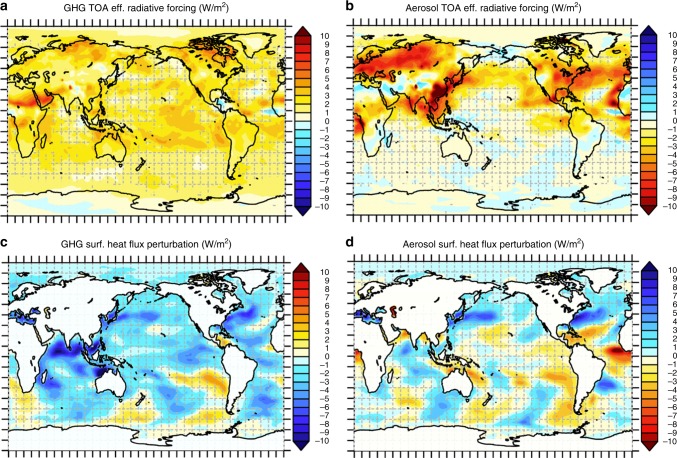
Spatial similarities in top-of-atmosphere (TOA) and surface forcing. **a**, **b** June–July–August (JJA)-mean TOA effective radiative forcing due to **a** greenhouse gases (GHGs) and **b** aerosols. **c**, **d** JJA-mean surface heat flux perturbation due to **c** GHGs and **d** aerosols. Note that the color palette for aerosol signals (**b**, **d**) is reversed relative to that for GHG signals (**a**, **c**). Gridded regions are not statistically significant at the 95% confidence level via *t*-test. See Supplementary Fig. 1 for December–January–February-mean values

We find that the top-of-the-atmosphere (TOA), all-sky, effective radiative forcings (ERFs)^6^ of GHGs and aerosols are weakly anti-correlated (*R* = −0.39) in our simulations, comparable to values found in other studies^3^ (Fig. 1). ERF is calculated as the difference in TOA radiative flux between simulations with and without the forcing agent after the atmospheric and land temperatures have been allowed to re-equilibrate GHGs’ TOA ERF pattern is by-and-large hemispherically symmetric and uniformly distributed (Fig. 2a, Supplementary Fig. 1a). Meanwhile, aerosols’ TOA ERF pattern (Fig. 2b, Supplementary Fig. 1b), due to aerosols’ short atmospheric lifetime, is dependent on regional factors like surface albedo and the location of emissions, and is concentrated in the Northern Hemisphere.

The TOA ERF patterns, however, are already subject to some homogenization through atmosphere and land adjustment to each forcer. The patterns of TOA ERF are, for example, substantially more spatially anti-correlated than are the aggregate concentrations of the forcers themselves (*R* = 0.03*). Although the concentrations of the forcers is not an appropriate proxy for the initial radiative effects of the two types of forcers^7^, it is valuable to note that the physical presence of the two forcers (as captured by their atmospheric concentrations) is entirely uncorrelated—highlighting that some homogenization occurs simply in how the climatological radiative environment interacts with the presence of a forcing agent.

Further, the TOA ERF does not represent only the initial radiative effects of the forcers, but also includes the radiative effects of microphysical, thermodynamic, and dynamical cloud responses to the presence of the aerosols or GHGs, as well as the outgoing longwave signature of the land surface temperature changes and associated lapse rate and albedo effects^6^. The spatial anti-correlation of the change in total cloud cover between the two runs (*R* = −0.43) is comparable to that for the all-sky TOA ERF, indicating that the latter can be explained partially by antisymmetric (i.e., symmetric, but of opposite sign) cloud changes in response to the two forcers. The spatially similar climatological cloud masking of aerosols’ and GHGs’ radiative effects may also play a role, as suggested by the slightly lower spatial correlation of the clear-sky (i.e., cloud non-permitting) TOA ERF (*R* = −0.40). Other radiative forcing definitions, such as instantaneous forcing, that are calculated prior to full atmospheric adjustment would not include such responses. However, such forcing definitions are impracticable for calculating aerosol radiative effects in models like AM3 that contain aerosol indirect effects on clouds and interactive translation of aerosol surface emissions into atmospheric concentrations—both of which require tropospheric adjustment in order to manifest.

### Spatially similar patterns of surface energy flux change

The spatial differences between the TOA ERF due to GHGs and that due to aerosols, however, are further homogenized in the surface energy flux perturbation due to each, achieving approximately two-thirds of the spatial similarity seen by Xie et al.^3^ in the eventual sea surface temperature (SST) response. The pattern of surface energy flux perturbation (Δ*S*, defined as the change in total surface energy flux, composed of radiative shortwave and longwave, latent, and sensible energy) in response to the two forcers is more strongly anti-correlated (*R* = −0.52) than the TOA ERF, indicating an additional process of homogenization between the TOA perturbation and the surface perturbation. Because the surface energy balance over land rapidly re-equilibrates due to the low effective heat capacity of the land surface, Δ*S* is near zero for all land surfaces by necessity. The spatial correlation of Δ*S* is therefore calculated only over the ocean, although the values are similar with and without land-masking. The oceanic surface energy balance is not constrained to re-equilibrate on atmosphere-only timescales, and as such will reflect the atmospheric conditions setting surface fluxes.

Δ*S* can be construed as an intermediary between the TOA atmospheric perturbation of a forcing agent and the ocean response thereto, and is thus a telling manifestation of the fast, atmosphere-and-land-only pathway for fully atmosphere-and-ocean coupled response pattern formation. The annual-mean, global-mean Δ*S* correlation between GHGs and aerosols emerges most strongly in the surface sensible heat flux component of the surface energy balance (Fig. 1). In contrast, the surface radiative (shortwave and longwave) flux change is little correlated (*R* = −0.13*), indicating that neither the radiative effects of climatological cloud masking nor of cloud change are responsible for the spatial similarities in Δ*S*. The strongest anti-correlation occurs largely during Northern Hemisphere summertime over the tropical and northern extratropical oceans, as indicated by the seasonal and latitudinal decomposition of Δ*S* (Fig. 1), and is driven by antisymmetric wave-like patterns in the surface latent and sensible heat flux change in response to each forcer (Fig. 2c, d).

### Similar atmospheric waves generated by land–sea contrast

**Fig. 3:**
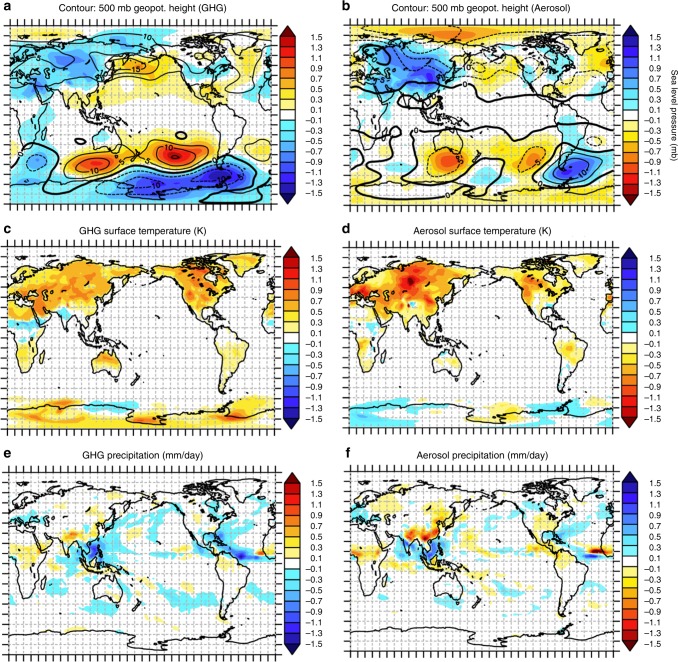
Spatially similar circulation changes. June–July–August (JJA)-mean perturbations due to **a**, **c**, **e** greenhouse gases (GHGs) and **b**, **d**, **f** aerosols in **a**, **b** 500 mb geopotential height (contour interval, 5 m) and sea level pressure, **c**, **d** surface temperature and **e**, **f** precipitation. Gridded regions are not statistically significant at the 95% confidence level via *t*-test. Note that the color palette for aerosol signals (**b**, **d**, **f**) is reversed relative to that for GHG signals (**a**, **c**, **e**). See Supplementary Fig. 2 for December–January–February-mean values

The common pattern of surface heat flux change in response to each forcer is a manifestation of a wave perturbation to the atmospheric circulation that is produced by both GHGs and aerosols. Over the extratropical oceans, a barotropic stationary Rossby wave perturbation to the atmospheric flow, consistent with the wave pattern in surface heat fluxes, is evident in alternating positive and negative anomalies collocated in sea level pressure (SLP) and in geopotential height at the 500-hpa pressure level (*Z*_500_) for both forcers (Fig. 3a, b, Supplementary Fig. 2a, b). The patterns of SLP and *Z*_500_ perturbations due to GHGs and aerosols are correlated at *R* = −0.44 and *R* = −0.71, respectively, during the Northern Hemisphere summertime when spatial correlation of Δ*S* is highest. Surface winds and temperature and specific humidity gradients between the surface and air, the primary controllers of surface heat fluxes, also exhibit strong wave patterns in the extratropics (Supplementary Figs. 3 and 4)—a consequence of the changes in extratropical atmospheric flow demonstrated in the SLP and *Z*_500_ anomalies. SLP and *Z*_500_ pattern correlations are weaker during the Southern Hemisphere summertime (*R* = −0.25* and *R* = −0.11*, respectively) and in the annual mean (*R* = −0.19* and *R* = −0.52, respectively), consistent with the seasonal dependence of the Δ*S* pattern correlation.

Rossby wave perturbations to the extratropical atmospheric flow can occur via wave sources located either in the extratropics or in the tropics. Wave excitation within the extratropics can result from changes in extratropical land–sea thermal and diabatic heating contrast caused by land surface temperature adjustments to forcing under the constraint of fixed SSTs^8,9^. Excitation of extratropical waves from within the tropics, meanwhile, can occur due to changes in tropical deep convection and precipitation (and thus atmospheric latent heating), the signal from which then propagates into the extratropics^10–13^.

GHGs and aerosols trigger these wave sources antisymmetrically. In the extratropics, the location of the landmasses serves as a potent fixed wave source in the presence of both forcers. The Northern Hemisphere summertime land–sea contrast (*T*_L/S_, quantified as the climatologically positive hemispheric-mean difference between land and ocean surface temperatures) decreases in the presence of aerosols (Δ*T*_L/S_ = −0.37 K) and increases in the presence of GHGs (Δ*T*_L/S_ = 0.35 K). This perturbation to the climatological land–sea contrast constitutes a diabatic heating anomaly that acts as an extratropical source of Rossby waves^8^. The surface temperature response to GHGs and aerosols (Fig. 3c, d) is constrained to the land in these prescribed SST runs. However, the land surface temperature response to the two forcers is spatially anti-correlated (*R* = −0.56, in the Northern Hemisphere summertime mean), likely constrained by local surface energy availability^14^ and regional sensitivities and feedbacks that are largely forcing independent^15^. The substantially weaker spatial correlation of surface energy flux patterns during the Northern Hemisphere winter-time (DJF) may be attributable to the higher variability and greater influence of transient eddies on stationary Rossby wave patterns during the Northern Hemisphere wintertime^16^, which decreases the influence of the land–sea contrast mechanism proposed here.

In the tropics, changes in deep tropical (20°S–20°N) precipitation due to GHGs and aerosols can also act as a source for extratropical Rossby waves. The fact that the simulated deep tropical precipitation changes are indeed antisymmetric (*R* = −0.54) (Fig. 3e, f) may contribute to the similar extratropical wave patterns. Interestingly, it is not straightforward why the two forcers would give rise to antisymmetric, rather than symmetric, precipitation changes. In fact, a thermodynamic scaling argument suggests the opposite: under fixed-SST conditions, both GHGs and aerosols increase tropospheric absorption of radiative energy, via increased absorption of longwave radiation in the case of GHGs and of shortwave radiation in the case of aerosols, and thus have suppressing effects on tropical mean precipitation^11,17–19^. Indeed, in our simulations, both forcers decrease tropical mean precipitation (by −1.2% for present-day GHGs and −0.74% for present-day aerosols).

In our view, the antisymmetric spatial pattern of these precipitation reductions is driven mainly by the opposite land–SST contrast patterns (Fig. 3c, d) and associated monsoonal circulation changes, which are dynamical in nature. The aerosol cools the land surface relative to the ocean, decreasing rainfall preferentially over the land surface, while the GHGs warm the land surface relative to the ocean, increasing the rainfall preferentially over the land surface. Analysis as part of the Precipitation Driver and Response Model Intercomparison Project finds similar behavior and mechanisms in the fast precipitation response to individual forcers^20^, as does analysis of future impacts of GHG and aerosol forcing in the CMIP5 archive^21^. Other, finer-scale features of the fast response to GHGs and aerosols that are evident in our simulations have also been confirmed in other analyses, such as weakening of the South Asian monsoon in the fast response to historical aerosol emissions^22^.

### The underlying driver of atmospheric energy redistribution

**Fig. 4:**
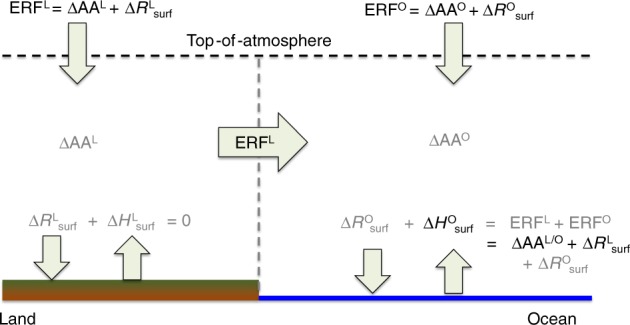
Energy balance constraints on surface heat flux patterns. The top-of-atmosphere (TOA) effective radiative forcings (ERF) over land and ocean (ERF^L/O^) must equal the surface radiative perturbation (∆*R*^L/O^_surf_) plus any atmospheric absorption (∆AA^L/O^) by vertical radiative energy conservation. Because the atmosphere and land surface cannot store energy long-term (*R*^L^_surf_ + *H*^L^_surf_ = 0, assuming positive downward sign convention), the TOA ERFs must be balanced through the ocean surface energy perturbation (*R*^O^_surf_ + *H*^O^_surf_). The ocean heat flux perturbation (*H*^O^_surf_) will, therefore, be responsible for balancing the total atmospheric absorption change plus the land surface radiative perturbation

Our above analysis of the mechanisms of surface pattern correlation demonstrates that the atmospheric circulation is an efficient homogenizer of heterogeneous forcings, even under fixed-SST conditions. Indeed, a simple energy balance analysis, based on energetic constraints alone, illuminates why this atmospheric homogenization must occur (Fig. 4). Because land surface and atmospheric energy perturbations rapidly equilibrate to zero, the total ocean surface energy perturbation (Δ*R*^O^_surf_ + Δ*H*^O^_surf_, with Δ*R* and Δ*H* denoting the radiative and heat components, respectively) must equal the TOA ERF over oceans (ERF^O^) plus that over land (ERF^L^) in the global mean: Δ*R*^O^_surf_ + Δ*H*^O^_surf_ = ERF^O^ + ERF^L^. Vertical longwave and shortwave radiative energy conservation dictates that the TOA ERF must equal the sum of the surface radiative perturbation (Δ*R*_surf_*)* and any net (i.e., absorption less emission) atmospheric absorption change (ERF = Δ*R*_surf_ + ΔAA). Thus, the surface heat flux perturbation over ocean will be equal to the total atmospheric absorption change over land and ocean plus the land surface radiative perturbation (Δ*H*^O^_surf_ = ΔAA^O+L^ + Δ*R*^L^_surf_), a balance that is evident in our simulations (Table 1). This coupling between the atmospheric and surface energetics over the land and the ocean dictates that most of the spatial heterogeneity in the initial TOA forcing cannot be maintained in the response and must be rapidly transformed (from radiation to heat) and redistributed (from the land surface and atmosphere to the ocean surface) by the atmospheric circulation. This circulation adjustment process is fundamentally responsible for the similar ocean heat flux perturbation patterns between GHGs and aerosols.

**Table 1:** Annual mean energy balance perturbations

Perturbation	Greenhouse gas	Aerosol
Ocean surf. heat flux (Δ*H*^O^_surf_)	0.73 (1.4)	−0.019 (−0.037)
Global atmos. abs. (ΔAA^O+L^)	0.64 (1.2)	0.41 (0.80)
Land surf. radiative flux (Δ*R*^L^_surf_)	0.092 (0.18)	−0.42 (−0.83)

Annual-mean perturbations to energy balance terms in Fig. 4 due to present-day greenhouse gases and aerosols in 10^15^ W are shown quantitatively (globally averaged in W/m^2^). Heat flux and radiative flux terms are positive downward, and atmospheric absorption terms are positive into the atmosphere

Notably, the fast homogenization described in this work operates primarily within a single hemisphere. The ratio of Northern Hemisphere to Southern Hemisphere TOA ERF does not differ significantly from that of the surface energy perturbation (1.0 and 1.1, respectively, for GHGs and 3.2 and 3.3, respectively, for aerosols). Under fixed-SST conditions, the Hadley circulation, the main mode of atmospheric cross-equatorial transport, cannot readily respond to perturbations^23^, constraining homogenization to within a given hemisphere.

## Discussion

Our work reveals that, even on the short timescales captured in fixed-SST simulations, GHGs and aerosols can be expected to produce strongly correlated spatial patterns of change across a range of variables. This is enforced by the symmetric perturbation to the extratropical circulation provided by land–sea contrast, the spatial structure of which is fixed by the location of the landmasses and, thus, relatively insensitive to the structure of the initial forcing. Analysis of the spatial correlation of atmosphere-and-land-only change in response to GHGs and aerosols in a range of models will be crucial to improved understanding of this phenomenon. We have here presented a picture of the atmosphere-and-land-only dynamical and thermodynamical mechanisms that drive these similarities in our model, providing a bridge to understanding the fully atmosphere-and-ocean coupled spatial patterns described by Xie et al.^3^ and others, and encourage continued analysis of this phenomenon in additional climate models.

The degree of similarity in the spatial pattern of the climate response to GHGs and aerosols has many implications for understanding and predicting the relative climate impacts of these forcers. Detection and attribution studies rely on spatial pattern as one component of the fingerprint of a given forcer, and similarity therein can result in a degradation of the ability to distinguish the signal from GHGs vs aerosols in climate phenomena^4^. Our identification here of additional climate variables in which spatial pattern similarity can be expected to emerge and the relatively rapid timescales on which they can be expected to do so (i.e., via decadal time-scale atmosphere and land adjustments) provide new inputs to determining spatial fingerprints for aerosols vs GHGs on the short-term timescales for which high quality, global observations are most available. A valuable extension to this work would be similar analysis of the role of atmosphere-and-land-only processes in setting the spatial pattern and fingerprint of response to scattering vs absorbing aerosols, which Xu and Xie^24^ have found to produce similar response patterns in fully coupled simulations.

Further, our understanding of the regional distribution of present and future climate change relies on constraining the spatial structure of the response to heterogeneous forcers like aerosols vs homogeneous forcers like GHGs^25,26^. Anthropogenic aerosols have been a primary mediator of GHG-driven climate change over the industrial era in the global mean^6^. Our findings indicate that the spatial distribution of the response to changes in global aerosol emissions will resemble that to GHGs even before slower coupled ocean processes kick in. This suggests that changes in global aerosol emissions may be capable of offsetting GHG-driven climate change on rapid, societally-relevant timescales not only in the global mean, but also in terms of the spatial distribution of climate change.

## Methods

### Atmospheric general circulation model simulations

All simulations in this study are conducted in the Geophysical Fluid Dynamics Laboratory’s AM3 Atmospheric General Circulation Model^27^ run with SSTs and sea ice prescribed from the Coupled Model Intercomparison Project 6 data set prepared for the Atmospheric Model Intercomparison Project^28^. The model contains 48 vertical layers in the atmosphere and has a cubed-sphere horizontal grid, with grid size varying from 163 to 231 km. AM3 contains fully interactive aerosols, which are transported and removed according to the internal meteorology and chemistry of the model. It simulates the first and second aerosol indirect effect in shallow convective and stratocumulus cloud, as well as internal mixing of black carbon and sulfate aerosol.

Three experimental set-ups are used, all run with natural forcings held fixed at 1860 values and with historically varying prescribed SST and sea ice: a 1860 forcing simulation with all anthropogenic and natural forcings held fixed at 1860 values (1860); an aerosol-only forcing simulation with historically varying anthropogenic aerosol emissions prescribed from Lamarque et al.^29^ (AERO); and a GHG-only forcing simulation with historically varying concentrations of well-mixed GHGs. All experimental set-ups are run with five ensemble members, and years 1970–2014 of each simulation are used in analysis. The GHG signals referenced in this work are derived as the difference between the GHG ensemble and a subset of the 1860 ensemble; the aerosol signals are derived as the difference between the AERO ensemble and an independent subset of the 1860 ensemble. Different 1860 ensemble members are used in the aerosol and the GHG calculations. The signals are thus entirely independent.

Statistical significances are calculated using a two-sided *t* test using the variability across model years, with sample size adjusted for autocorrelation between model years^30^. It should be noted that the AM3 model has particularly high internal variability compared to other climate models included in the Fifth Coupled Model Intercomparison Project^31^.

### Correlation coefficients

Pearson correlation coefficients throughout are calculated via linear regression with area-weighting. Scatterplots of all data were analyzed to ensure insensitivity to outliers and qualitative linearity of relationships. Correlation values given are significant at the 95% level under a two-sided *t* test, except where they are followed by an asterisk (*).

### Code availability

The code for the GFDL AM3 model is publicly available at https://www.gfdl.noaa.gov/am3/. The code used to set up the simulations used in this study and to analyze data is available from the authors upon request.

### Data availability

All data analyzed in this work is accessible at ftp://ftp.gfdl.noaa.gov/pub/ggp/Persadetal_files/. This includes the raw data associated with the generation of Figs. 1–3 and Supplementary Figures 1–4.

## Electronic supplementary material

Supplementary Information

## References

[1] Levy H, Schwarzkopf MD, Horowitz L, Ramaswamy V, Findell KL (2008). Strong sensitivity of late 21st century climate to projected changes in short-lived air pollutants. J. Geophys. Res..

[2] Levy H (2013). The roles of aerosol direct and indirect effects in past and future climate change. J. Geophys. Res. Atmos..

[3] Xie SP, Lu B, Xiang B (2013). Similar spatial patterns of climate responses to aerosol and greenhouse gas changes. Nat. Geosci..

[4] Bindoff, N. L. et al. In *Climate Change 2013: The Physical Science Basis. Contribution of Working Group I to the Fifth Assessment Report of the Intergovernmental Panel on Climate Change *(eds Stocker, T. F. et al.) Ch. 10 (Cambridge University Press, 2013).

[5] Shindell DT (2014). Inhomogeneous forcing and transient climate sensitivity. Nat. Clim. Change.

[6] Myhre, G. et al. In *Climate Change 2013: The Physical Science Basis. Contribution of Working Group I to the Fifth Assessment Report of the Intergovernmental Panel on Climate Change* (eds Stocker, T. F. et al.) Ch. 8 (Cambridge University Press, 2013).

[7] Xia Y, Huang Y (2017). Differential radiative heating drives tropical atmospheric circulation weakening. Geophys. Res. Lett..

[8] Held IM, Ting M, Wang H (2002). Northern winter stationary waves: theory and modeling. J. Clim..

[9] Ming Y, Ramaswamy V (2011). A model investigation of aerosol-induced changes in tropical circulation. J. Clim..

[10] Sardeshmukh P, Hoskins B (1987). The generation of global rotational flow by steady idealized tropical divergence. J. Atmos. Sci..

[11] Held IM, Soden BJ (2006). Robust responses of the hydrological cycle to global warming. J. Clim..

[12] Vecchi GA, Soden BJ (2007). Global warming and the weakening of the tropical circulation. J. Clim..

[13] Ming Y, Ramaswamy V (2009). Nonlinear climate and hydrological responses to aerosol effects. J. Clim..

[14] Andrews T, Forster PM, Gregory JM (2009). A surface energy perspective on climate change. J. Clim..

[15] Armour KC, Bitz CM, Roe GH (2012). Time-varying climate sensitivity from regional feedbacks. J. Clim..

[16] Ting M, Wang H, Yu L (2001). Nonlinear stationary wave maintenance and seasonal cycle in the GFDL R30 GCM. J. Atmos. Sci..

[17] Roeckner E, Bengtsson L, Feichter J, Lelieveld J, Rodhe H (1999). Transient climate change simulations with a coupled atmosphere–ocean GCM including the tropospheric sulfur cycle. J. Clim..

[18] Allen MR, Ingram WJ (2002). Constraints on future changes in climate and the hydrologic cycle. Nature.

[19] Ming, Y., Ramaswamy, V. & Persad, G. Two opposing effects of absorbing aerosols on global-mean precipitation. *Geophys. Res. Lett*. **37**, L13701 (2010).

[20] Samset BH (2016). Fast and slow precipitation responses to individual climate forcers: a PDRMIP multimodel study. Geophys. Res. Lett..

[21] Tian, D., Dong, W., Gong, D., Guo, Y. & Yang, S. Fast responses of climate system to carbon dioxide, aerosols and sulfate aerosols without the mediation of SST in the CMIP5. *Int. J. Climatol*. **37**, 1156–1166 (2016).

[22] Ganguly D, Rasch PJ, Wang H, Yoon J (2012). Fast and slow responses of the South Asian monsoon system to anthropogenic aerosols. Geophys. Res. Lett..

[23] Hill, S. A., Ming, Y. & Held, I. M. Mechanisms of forced tropical meridional energy flux change. *J. Clim*. **28**, 1725–1742 (2015).

[24] Xu Y, Xie SP (2015). Ocean mediation of tropospheric response to reflecting and absorbing aerosols. Atmos. Chem. Phys..

[25] Shindell D, Faluvegi G (2009). Climate response to regional radiative forcing during the twentieth century. Nat. Geosci..

[26] Shindell, D. et al. Spatial scales of climate response to inhomogeneous radiative forcing. *J. Geophys. Res. Atmos.* **115**, D19110 (2010).

[27] Donner LJ (2011). The dynamical core, physical parameterizations, and basic simulation characteristics of the atmospheric component AM3 of the GFDL global coupled model CM3. J. Clim..

[28] Taylor, K. E., Williamson, D. & Zwiers, F. *The Sea Surface Temperature and Sea Ice Concentration Boundary Conditions for AMIP II Simulations* 25 (Lawrence Livermore National Library, 2000).

[29] Lamarque JF (2010). Historical (1850–2000) gridded anthropogenic and biomass burning emissions of reactive gases and aerosols: methodology and application. Atmos. Chem. Phys..

[30] Santer BD (2000). Statistical significance of trends and trend differences in layer-average atmospheric temperature time series. J. Geophys. Res. Atmos..

[31] Knutson TR, Zhang R, Horowitz LW (2016). Prospects for a prolonged slowdown in global warming in the early 21st century. Nat. Commun..

