# Supplementary material for: Spatially similar surface energy flux perturbations due to greenhouse gases and aerosols
Source: Nat Commun. 2018 Aug 14;9:3247. doi: 10.1038/s41467-018-05735-y (PMC6092435; doi:10.1038/s41467-018-05735-y)
Supplement: Supplementary file 1 — Supplementary Information [file 41467_2018_5735_MOESM1_ESM.pdf]

Supplement to Spatially Similar Surface Energy Flux Perturbations due to Greenhouse Gases and Aerosols

Persad et al.

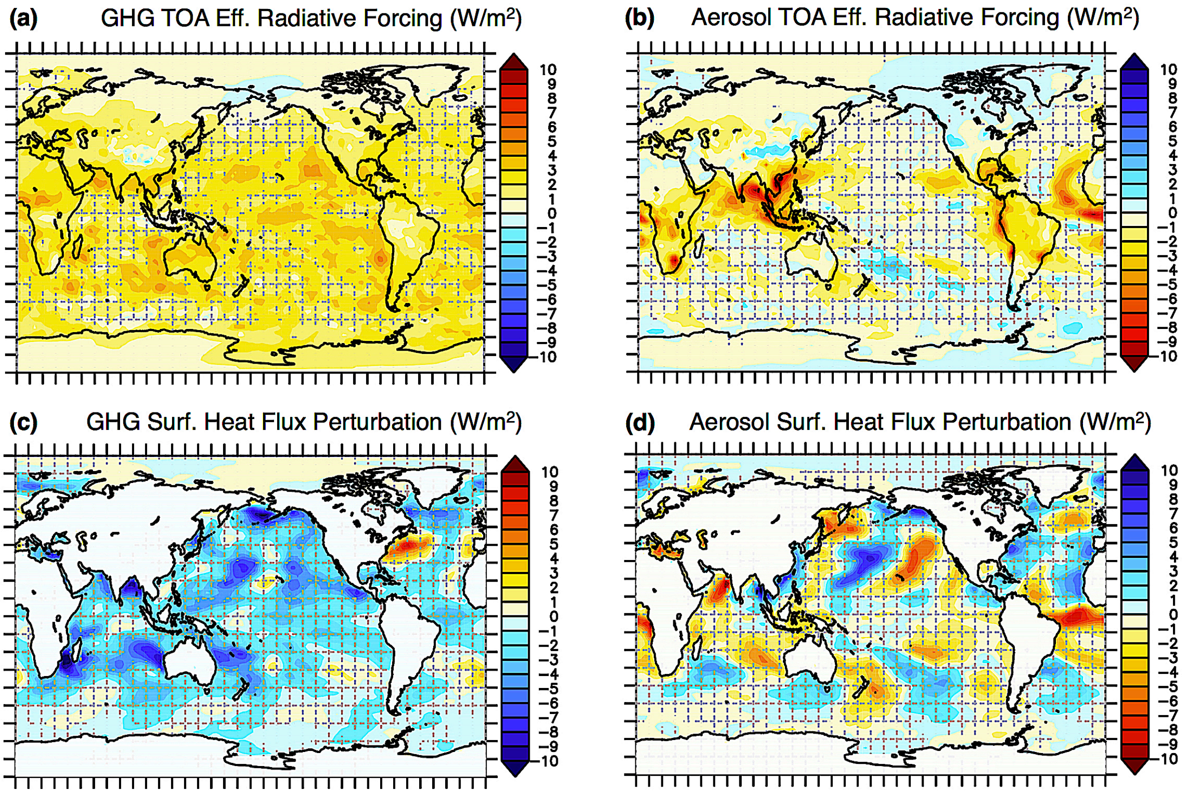

Supplementary Figure 1 | Spatial Similarities in TOA and Surface Forcing. (a, b) DJF-mean TOA Effective Radiative Forcing due to **(a)** GHGs and (**b**) aerosols. **(c, d)** DJF-mean surface heat flux perturbation due to **(c)** GHGs and (**d**) aerosols. Gridded regions are not statistically significant at the 95% confidence level via t-test. Note that the color palette for aerosol signals **(b, d)** is reversed relative to that for GHG signals **(a, c).**

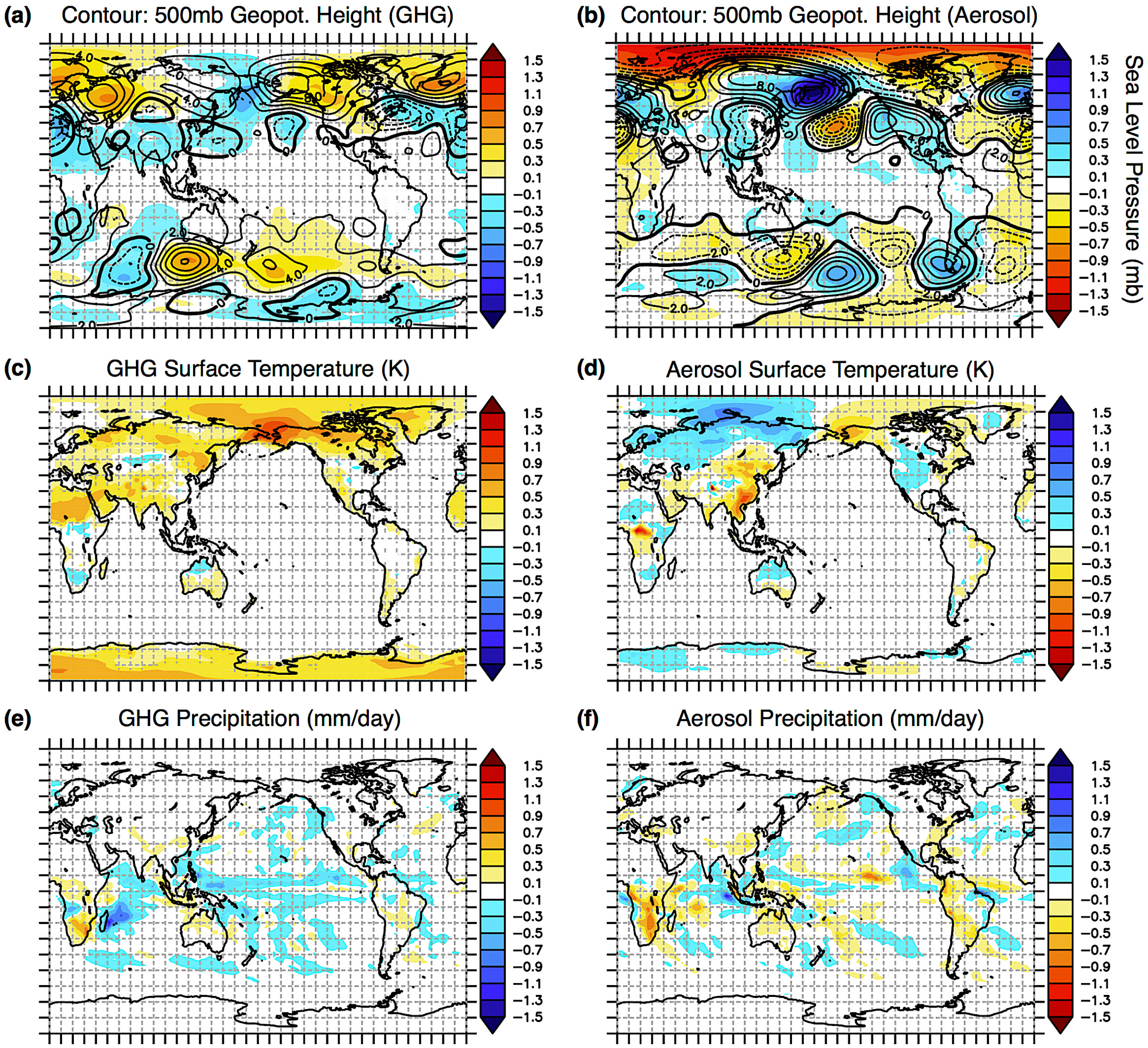

Supplementary Figure 2 | Spatially similar circulation changes. DJF-mean perturbations due to **(a, c, e)** GHGs and **(b, d, f)** aerosols in **(a, b)** 500 mb geopotential height (contour interval, 5 m) and sea level pressure, **(c, d)** surface temperature and **(e, f)** precipitation. Gridded regions are not statistically significant at the 95% confidence level via t-test. Note that the color palette for aerosol signals **(b, d, f)** is reversed relative to that for GHG signals **(a, c, e).**

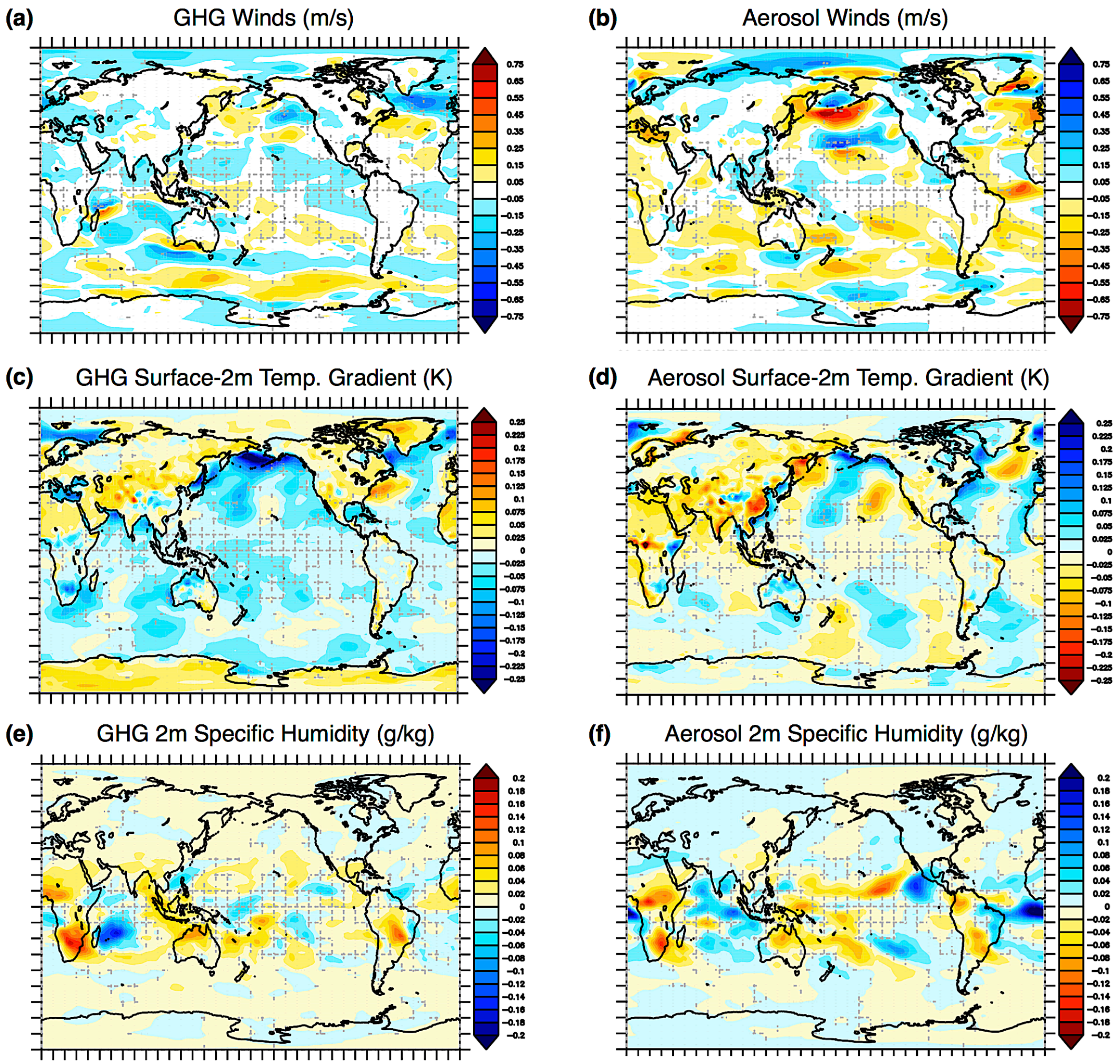

Supplementary Figure 3 | DJF Spatial Similarities in Surface Heat Flux Drivers. DJF-mean perturbations due to **(a, c, e)** GHGs and **(b, d, f)** aerosols in **(a, b)** 2m wind speed (m/s, northward positive), **(c, d)** surface-2m temperature gradients (K) and **(e, f)** 2m specific humidity (g H_2O/kg air). Gridded regions are not statistically significant at the 95% confidence level via t-test. Note that the color palette for aerosol signals **(b, d, f)** is reversed relative to that for GHG signals **(a, c, e).**

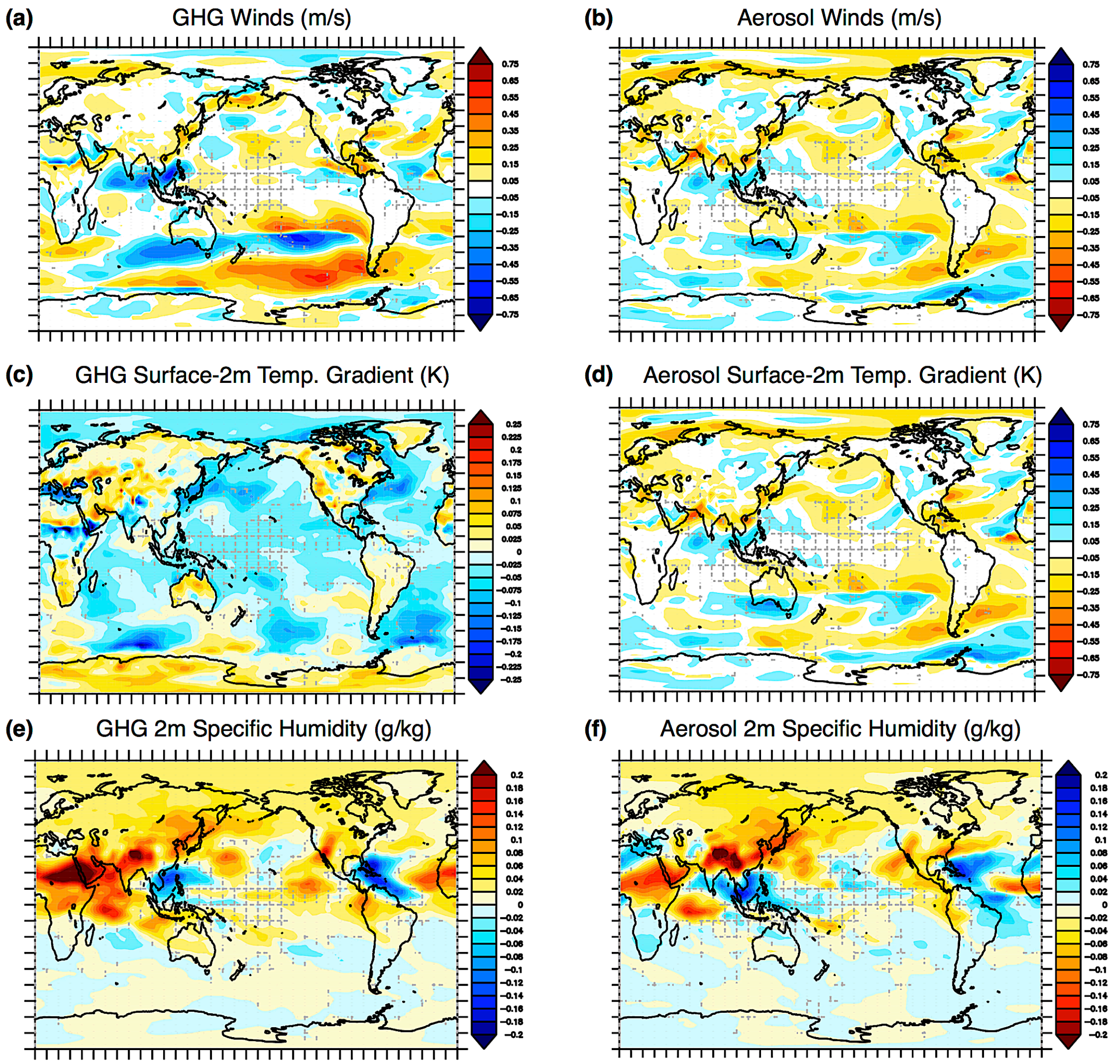

Supplementary Figure 4 | JJA Spatial Similarities in Surface Heat Flux Drivers. JJA-mean perturbations due to **(a, c, e)** GHGs and **(b, d, f)** aerosols in **(a, b)** 2m wind speed (m/s, northward positive), **(c, d)** surface-2m temperature gradients (K) and **(e, f)** 2m specific humidity (g H_2O kg_{air}^{-1}). Gridded regions are not statistically significant at the 95% confidence level via t-test. Note that the color palette for aerosol signals **(b, d, f)** is reversed relative to that for GHG signals **(a, c, e).**
